# Recent Advances and Review on Treatment of Stiff Person Syndrome in Adults and Pediatric Patients

**DOI:** 10.7759/cureus.427

**Published:** 2015-12-22

**Authors:** Adnan Bashir Bhatti, Zarine Anwar Gazali

**Affiliations:** 1 Department of Medicine, Capital Development Authority Hospital, Islamabad, Pakistan; 2 Research Fellow, MITR Hospital, Kharghar, Navi Mumbai, Maharashtra, India

**Keywords:** stiff person syndrome, autoimmune diseases, neurological disorders, glutamic acid decarboxylase antibody, stiff man syndrome

## Abstract

Stiff Person Syndrome (SPS) is one of the rarest autoimmune neurological disorders, which is mostly reported in women. It is characterised by fluctuating muscle rigidity and spasms. There are many variants of SPS, these include the classical SPS, Stiff Leg Syndrome (SLS), paraneoplastic variant, gait ataxia, dysarthria, and abnormal eye movements. Studies have shown that the paraneoplastic variant of SPS is more common in patients with breast cancer who harbour amphiphysin antibodies, followed by colon cancer, lung cancer, Hodgkin's disease, and malignant thymoma.

Currently, the treatment for SPS revolves around improving the quality of life by reducing the symptoms as far as possible with the use of GABAergic agonists, such as diazepam or other benzodiazepines, steroids, plasmapheresis, and intravenous immunoglobulin (IVIG). There have been random clinical trials with Rituximab, but nothing concrete has been suggested. A treatment approach with standard drugs and cognitive behavioral therapy (CBT) seems to be promising.

## Introduction and background

Stiff Person Syndrome (SPS) dates back to as long as 1956 where Moersch and Woltman first described the tightness of the back, abdominal, and thigh muscles in 14 patients. They further conducted a study for a period of 32 years to conclude their findings of progressive fluctuating, rigid, and painful spasms that lead to a wooden man appearance as SPS [1]. Almost a decade later, Howard first reported the use of diazepam, which gave relief to SPS-associated symptoms [2]. Major benchmarks were achieved in 1988 when anti-glutamic acid decarboxylase (anti-GAD) antibodies were discovered in SPS, and consequently, corticosteroids were used to manage SPS symptoms. The results were promising and, hence, it was put forth as a new treatment modality. In the past few decades, extensive research on plasmapheresis, intravenous immunoglobulin (IVIG), and various antibodies allowed their introduction in the management of SPS. The link between anti-amphiphysin, anti-gephyrin, anti-GABA_A_ receptor associated protein (anti-GABARAP), and paraneoplastic SPS were also discovered [3-4]. 

The exact pathophysiology of SPS still remains unclear, but the widely accepted theory is that of the involvement of anti-GAD, which are a group of cytoplasmic enzymes involved in GABA synthesis in brain and spinal cord [5]. There are classically two isoforms of anti-GAD: GAD65 and GAD67. The former is associated to SPS, diabetes mellitus, cerebellar ataxia, and limbic encephalitis [6-8].

**Figure 1:**
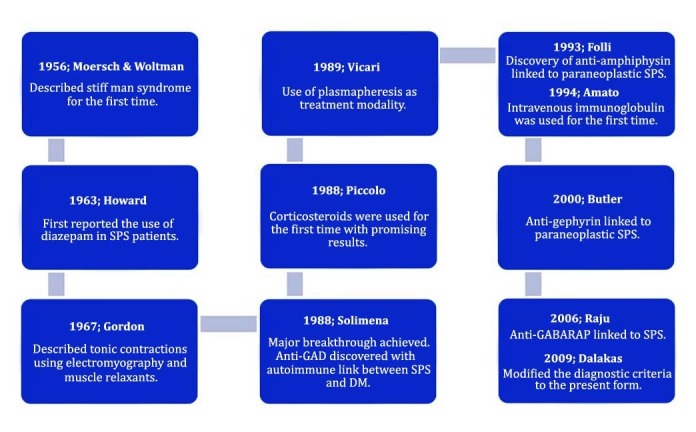
Major achievements that contributed to Stiff Person Syndrome (SPS) treatment and research.

The incidence of SPS is very rare and the prevalence of the disease is one in a million [9]. SPS cases are difficult to diagnose owing to their rarity and, hence, about 60% of the cases get diagnosed only because of the presence of anti-GAD65 in the blood [10]. The GAD and amphiphysin are both presynaptic autoantigens while GABARAP and gephyrin are postsynaptic autoantigens [11-13]. In SPS, there is no structural damage seen to the GABAergic neurons and the pathology is presumed to be due to a pharmacological blockade. There are no neurological symptoms seen in SPS, besides an increase in muscle tone. This is backed up by the normal post-mortem findings and improved symptoms with immunotherapy [14-15]. Major achievements that have contributed to SPS research are as given in Figure 1.

### Clinical presentation

SPS is a rare disorder and, therefore, a neurologist may encounter just one or two cases during his/her entire clinical practice. Patients may have an insidious onset with classical findings being episodic aching and stiffness of the axial muscles slowly progressing to proximal muscles. As the disease progress, the patients may find it difficult to carry out their day-to-day activities. Clinical symptoms present themselves at a mean age of 41.2 years (range: 29-59 years). Neonatal cases are also reported very rarely.

The common features seen in SPS include:

1. Stiffness starting in the trunk and progressing to the abdomen and lumbar region. Hyperlordosis due to the episodic aching and stiffness of the lumbar spine is a diagnostic hallmark of SPS [16]. 2. The stiffness progresses to other muscles in the body, for instance, progression to the thorax muscles causing breathing difficulties. Facial muscle involvement gives an emotionless, mask-like appearance [15]. 3. Painful spasms are elicited by triggers predominantly auditory or tactile in origin, and they are in sync with those observed in the case of tetanus. 4. Joint dislocations and fracture have been observed in some cases with the sudden onset of spasm. 5. Normal sensation, motor function, and intellect are present. 6. An association with psychological disorders is also seen [15]. 7. Electromyographic (EMG) findings are supportive of continuous motor activity. 8. Serology testing positive for GAD65 autoantibodies.

**Figure 2:**
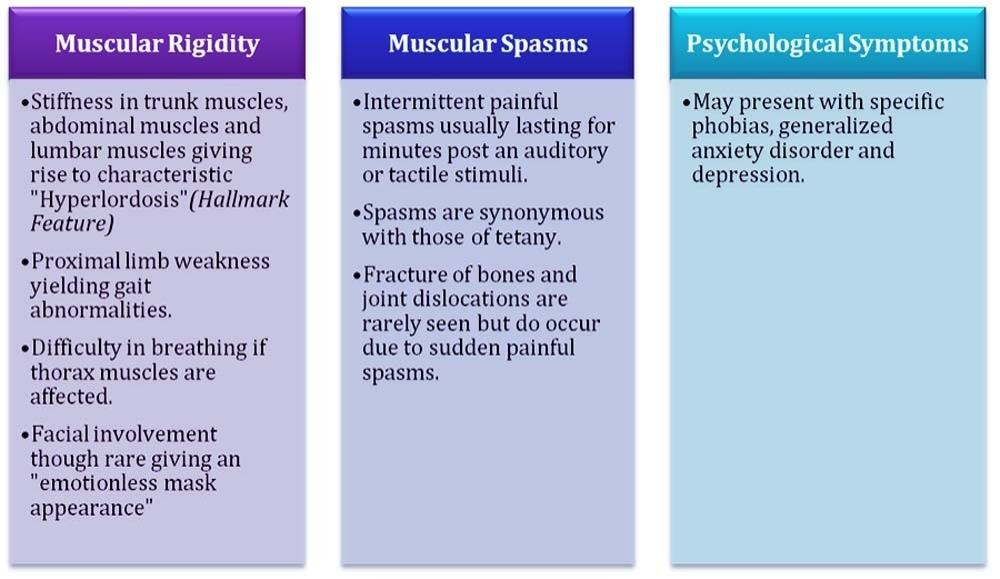
Clinical features of Stiff Person Syndrome (SPS).

Continuous muscle fibre activity on EMG and anti-GAD are pathognomic of SPS. Anti-amphiphysin, anti-GABARAP, and anti-gephyrin may be present in the patient’s serum or CSF in GAD-negative patients. For more clarity, the clinical feature of SPS is summarized in Figure 2.

## Review

The prime focus in SPS is aimed at giving symptomatic relief to the patient and improving the quality of life. Due to the rarity of the disease, there are limitations in the quality of treatment options that are available. The past few decades have thrown some light on various approaches for reducing the spasticity and rigidity of muscles in SPS. The discovery of anti-GAD proved to be the most important pathognomic finding in SPS.

Over the years, treatment modalities for SPS have included benzodiazepines and baclofen as the first line of drugs followed by IVIG, plasmapheresis, immune modulators, and Rituximab. IVIG and plasmapheresis are either used alone or in combination in refractory cases.

Corticosteroids are used as monotherapy or in combination with other drugs for SPS. However, their efficacy is not determined by any clinical trials. The paraneoplastic variant of SPS, where stiffness is localized to the arms and legs, makes up to just 5% of SPS cases. Classical SPS patients respond well to treatment, but in about 10% of cases, sudden deaths occur due to autonomic dysfunction [16]. Repeated spasms or sudden withdrawal of medicine may lead to autonomic dysfunction, resulting in sudden death [17].

### Benzodiazepine as first line drug

Benzodiazepines are considered as the first line treatment in patients diagnosed with SPS. Diazepam, being a GABA_A_ agonist, is not only used as an anticonvulsant but is also used in SPS management owning to its profound muscle relaxant property. A divided dose of 5-100 mg of diazepam or clonazepam (divided dose 1-6 mg) are given by gradually increasing the dose over time [17]. The administration of higher doses at the beginning of treatment may make patients susceptible to dangerous adverse effects, including respiratory depression along with drowsiness and dysarthria.

### Other GABAergic drugs

Other drugs, such as gabapentin, tiagabine, valproate, and levetiracetam, have been used for reducing the SPS symptoms. Vigabatrin was used in the past but now has been discontinued due to its probable side-effect of causing visual field constriction. Levetiracetam (2000 mg) was tested in a single, blind placebo controlled trial in just three patients and showed reduced the symptoms in SPS [18].

### Oral baclofen vs intrathecal baclofen

Baclofen is mainly used orally, along with diazepam, as a first line treatment for its GABA_B_ agonist activity to manage spasticity. Due to its low CSF bioavailability, intrathecal baclofen (50-800 µg/day) has been used to treat severe spasticity, which has shown significant improvement in symptoms of SPS. However, utmost care must be taken as chances of catheter infection, catheter leakage, pump failure, and, in some cases, death may occur due to autonomic failure [19-21].

### Treatment with plasmapheresis over intravenous immunoglobulin - the better approach

As per the European Federation of Neurological Societies (EFNS), IVIG (2 g/kg over two to five days) should be reserved for patients who have no symptomatic relief after the use of diazepam and/or baclofen and have a severe disability in carrying out daily activities [22]. The result of a randomised, double blinded, placebo-controlled, crossover trial on patients treated with IVIG has showed improvement in their symptoms with a significant decrease in stiffness and decrease in GAD autoantibodies [23]. The GAD autoantibody titre also decreased after administration of IVIG [23].

IVIG is usually safe but has higher chances of adverse reactions as compared to plasmapheresis, ranging from mild to severe in patients with IgA deficiency and, hence, is contraindicated in them. On the other hand, plasmapheresis therapy has shown promising results in 56% patients registered in the study approved by John Hopkins Institute (JHH) where first-line treatment failed [24]. Studies have shown that plasmapheresis is well tolerated with adverse effects seen in just 4.75% of patients receiving it [25].

### Treatment approach based on presence of GABARAP, GAD, GlyRα1, and amphiphysin antibodies 

It is observed that the anti-GAD autoantibodies have been associated with involvement of trunk, abdominal, and limb muscles. However, 80% of SPS patients who tested positive for amphiphysin have shown a strong association with rigidity in cervical muscles and were paraneoplastic [26]. In recent times, immense research has been done to identify the autoantigens. It had led to evidence that GABARAP; which is a 14-kD protein localized at postsynaptic region of GABA-ergic synapses, inhibits GABA_A_ receptor expression in about 65% of SPS patients. Such patients have responded better to IVIG as opposed to high doses of GABA-enhancing drugs that cause undesirable adverse effects [27].

Patients with amphiphysin antibodies are known to respond better to steroids, plasmapheresis, or treatment of the primary cause (e.g. breast cancer) while those with anti-GAD responded well to IVIG, diazepam (37 mg/day), and clonazepam (4mg/day) [28-29]. Patients with GlyRα1 antigen respond better to immunotherapies than patients with GAD65 immunoglobulin.

### Promising prospects of Rituximab

Rituximab, a monoclonal antibody that binds to the B-lymphocyte cluster of differentiation (CD) surface antigen, has been tried as an effective drug to manage SPS. It is administered as at least two doses each of 350-375 mg/ m^2^^ ^infusion with a spacing of seven to 14 days or as four weekly infusions, which have resulted in a substantial decrease in the severity of symptoms [30].

After the failure of benzodiazepines and monthly IVIG treatment, marked gait improvement and ambulation with minimal assistance were achieved after the administration of two doses of rituximab, each of 500 mg/m^2 ^spaced over 14 days [31]. For relapse cases with anti-GAD positivity in the serum or CSF, repeat doses six to eight months later have reported to be favourable [32].

Though very few papers have reported the effective use of rituximab, it should still be considered as an alternative treatment for patients with SPS when the treatment with benzodiazepines and other conventional antispasmodic immunotherapies have failed to produce the desired effect [33].

### Cognitive behavioral therapy

Muscle stiffness gets exaggerated due to anxiety as it is an autonomic physiological symptom. A study conducted have showed that about 44% of the patients develop severe motor symptoms due to their anxiety [34]. A case study was conducted on an SPS patient who underwent five weeks of CBT. The results were promising as evidenced by the substantial decrease in anxiety, upliftment of the self-confidence, and lessening stiffness and rigidity [35].

### Pediatric approach in management of SPS

Since SPS is a very rare disorder in adults and manifests later in life, diagnosis of SPS at a pediatric age is very challenging. It may quite resemble tetanus in presentation and thus often lead to misdiagnosis. Tetanus follows an acute course with recovery in few weeks while SPS is a chronic disorder with varying degrees of disability, which does not improve over time [36]. Though unusual features like mild trismus and blepharospasm point to tetanus, the overall time period should be taken into account and other clinical features should be ruled out to confirm the diagnosis of tetanus [36].

The pathophysiology in childhood SPS is still unclear as compared to that of adults. Childhood SPS often demonstrates GlyRα1 mutation. Lately, there has been a strong correlation with striatal lesions and childhood SPS in contrast to spinal and brain lesions in adult SPS [37]. Most children with SPS also have negative anti-GAD and exhibit acute onset with a transient benign course [38]. They may also be associated with psychiatric disorders but frequently go unnoticed. No prospective clinical study has been carried out to outline specific modalities targeting the pediatric group due to the ameliorated data.

Neonates may also present with SPS immediately after birth. The clinical features include an exaggerated startle response, rigidity, and acquisition of flexed fetal position. The hallmark symptom is flexor spasm in response to a light tap on the nose. If left untreated, it leads to sudden death in sporadic cases due to severe spasm [39]. Delayed motor milestones with low intelligence have also been observed [40-41].

Benzodiazepines, the classical first line drugs for SPS, are used for treating childhood SPS as well. Benzodiazepines given intravenously, along with IVIG, have shown gradual significant improvement [42]. However, due to the limitations of insufficient work in this field, nothing conclusive can be derived and more research in this field is required.

## Conclusions

SPS is a rare disorder and is very difficult to diagnose. With a timely recognition of the disease and prompt treatment, the quality of life of SPS patients can be improved. Though the first line of drugs for SPS is benzodiazepines and baclofen, their dose-related adverse effects are of major concern. Intrathecal baclofen is a better alternative but care should be practiced to avoid complications, such as infection via the catheter. An improved clinical study focusing combination therapy for SPS may prove beneficial. A combination therapy of benzodiazepines with CBT and IVIG or plasmapheresis, depending on the type of antibody, can be chosen for managing SPS. Less data is available on the pediatric onset of SPS, besides a few handpicked case reports, therefore, making it hard to be conclusive on an effective treatment option. In general, research on SPS is very limited, largely owing to the rarity of the disease. Therefore, more research should be done in this field, which may in turn help patients, although low in number, from the debilitating effects of SPS.
